# Preceptorship in robotic colorectal surgery: experience from the Australian private sector

**DOI:** 10.1007/s11701-024-01972-0

**Published:** 2024-05-17

**Authors:** Chahaya Gauci, Assad Zahid, Praveen Ravindran, Andrew Craig Lynch, Stephen Pillinger

**Affiliations:** 1 Australian Robotic Colorectal Surgery, Sydney Adventist Hospital, Sydney, NSW Australia; 2 University of Sydney (Sydney Medical School), Sydney, NSW Australia; 3 St George and Sutherland Clinical School, University of New South Wales Medicine, Sydney, NSW Australia; 4 College of Health and Medicine, Australian National University, Canberra, ACT Australia

**Keywords:** Robotic surgical procedures, Colorectal surgery, Education, Professional, Preceptorship, Competency-based education

## Abstract

This article describes a post-fellowship preceptorship training program to train sub-specialty colorectal surgeons in gaining proficiency in robotic colorectal surgery using a dual-surgeon model in the Australian private sector. The Australian colorectal surgeon faces challenges in gaining robotic colorectal surgery proficiency with limited exposure and experience in the public setting where the majority of general and colorectal surgery training is currently conducted. This training model uses graded exposure with a range of simulation training, wet lab training, and clinical operative cases to progress through both competency and proficiency in robotic colorectal surgery which is mutually beneficial to surgeons and patients alike. Ongoing audit of practice has shown no adverse impacts.

## Introduction: history of surgical training

In the rapidly evolving landscape of surgical care, the advent of robotic technology represents a paradigm shift, necessitating a re-evaluation of traditional surgical training methodologies. The transition from traditional minimally invasive surgical techniques to robotic-assisted procedures underscores not only advancements in healthcare technology but also the critical need for specialized training programs. These programs will need to be designed to equip the next generation of surgeons with the proficiency required for robotic colorectal surgery, ensuring that they are not only adept at utilizing these advanced technologies but also capable of pushing the boundaries of minimally invasive surgery. This evolution highlights the importance of integrating robotic surgery training into post-fellowship education, reflecting a commitment to patient safety, surgical excellence, and the continuous advancement of surgical practice driven by master surgeons.

The history of surgical training is a testament to human progress in medicine and technology. Ancient civilizations relied on informal apprenticeships for surgical education. In the Middle Ages, the first formal medical schools including the Schola Medica Salernitana, began to take shape and established a more structured approach including the use of textbooks to pass on standardized approaches to surgery [1].

The Renaissance brought significant advancements in surgical techniques, fueled by the pioneering work of individuals like Ambroise Paré [2]. The nineteenth century saw the transformative impact of anesthesia, revolutionizing surgery, and expanding its possibilities.

The twentieth century marked a turning point with the standardization of surgical training, the establishment of residencies, and specialization. In the United States, Halsted built upon European models and established rigorous surgical residencies at John Hopkins Hospital, fostering the development of a more holistic surgeon who was not only technically skilled but also encouraged aspiring surgeons to be lifelong students, surgeon scientists, and in turn surgeon educators [3]. He is oft quoted and associated with his ‘see one, do one, teach one’ [4]. Ultimately, the Flexner Report of 1910 elevated medical education standards, emphasizing scientific rigor [5].

Post-graduate training and interprofessional collaboration with a focus on comprehensive patient care has since continued to evolve and gained prominence. Throughout history, the unwavering commitment to patient safety and excellence in surgical training continues to shape the future of surgery, ensuring better outcomes and improved care. Throughout the international surgical community, various Colleges of Surgeons have established educational programs with a theoretical and skills-based curriculum. More recently there has also been a move to competency-based acquisition of skills rather than absolute time-based training. In Australia, two of the surgical specialty training programs have moved to competency-based training with flexibility in the absolute time required [6].

The question arises then how do established surgeons acquire new skills, techniques or adopt new technology? Two major advances in surgical approach which have required established surgeons to establish new skills are laparoscopic surgery and subsequently robotic surgery.

Advancements in video technology and minimally invasive surgery (MIS) and techniques further refined surgical training. In the last decade, cutting-edge innovations like high-fidelity surgical simulations and robotic-assisted surgery revolutionized how trainees can acquire skills [7]. Robotic devices used for MIS can run simulations, provide objective feedback on performance indicators such as excessive force, instruments out of view, economy of motion, instrument collisions, master workspace range or number of drops. As Ericsson highlighted, it is not simply the social construct of an expert but objective measurements of expertise that are important and finally this can be facilitated in surgery with much greater ease [8]. Beyond this, robotic surgery can allow dual console operating in a student-master setup, allowing safe supervision, direction for progressing intra-operatively and handover as required between operative steps without interruption to standard operative flow [9].

Skill acquisition and education has innumerable theories and principles, too vast to detail in their entirety; however, in established formal surgical education, there are some fundamental principles to consider [10]. ‘Cognition’, ‘integration’, ‘automation’, and an extension to this, the “after”, are concepts by Hamdorf and Hall that are oft referred to and expanded upon [10, 11]. The original description of the first of the phases, ‘cognition’, describes a focus on perceptual awareness, or development of an understanding of the concepts and steps involved. This was followed by translation into the mechanical components involved, ‘integration’, then refined with improvements in speed, efficiency, and precision: ‘automation’ [11]. The concept of surgical skill acquisition emphasizes the importance of technical learning being consolidated through deliberate practice—this part may be referred to as the ‘after’ [7, 10]. This practice is a structured and intentional process aimed at improving performance in a specific area. It requires focused and effortful repetition of skills in progressive exercises, accompanied by immediate, informative feedback tailored to the specific task—or simply coaching, and may involve simulation [7, 8, 10]. This feedback is crucial for trainees as it aids in refining their skills and adapting their techniques more effectively. Robotic preceptorship and the program described below largely focuses on this final component.

When establishing the desired outcome, there must be a common definition of goals. Louridas and de Montbrun who have published extensively on surgical education have outlined the importance of differentiating between competency and proficiency [12]. Competency denotes the foundational level of skill necessary to execute a task safely and without supervision, whereas proficiency indicates a more advanced skill level, approaching mastery and exceeding mere competency [12, 13]. These concepts were supported in a systematic review by Szasz et al., with technical competence having the common theme of a ‘minimum standard’ versus proficiency indicating a more advanced skillset [13].

## Laparoscopic surgical training and adoption

The ‘Lapco program’ (Laparoscopic Colorectal Surgery Training Program) was a significant initiative for colorectal surgeons in the United Kingdom. Launched in 2008, it was designed to promote uptake of laparoscopic techniques for colorectal surgery by providing teaching to experienced and established colorectal surgeons through competency-based training [14]. The program provided specialized training through workshops, courses, and mentorship during operative cases. The program resulted in a wider adoption of laparoscopic techniques among colorectal surgeons and competency-based training has improved surgeons’ skills, resulting in better patient outcomes, reduced complications, and shorter hospital stays [14]. The “train the trainer” approach has established a network of skilled laparoscopic surgeons who can mentor and train others, ensuring the sustainable development and dissemination of expertise. The program’s emphasis on mentorship and hands-on training fosters a collaborative learning environment, promoting knowledge sharing and best practices. As a result, Lapco has played a pivotal role in advancing colorectal surgery in the UK, introducing innovative techniques, and raising the overall standard of care for patients [14].

## Robotic surgical training in Australia

Robotic surgery is the latest paradigm shift but uptake in Australia remains a gradual progress. Unfortunately, in Australia, robotic surgery has been limited by perceived and real cost constraints, how this fits within the Australian public hospital system, as well as initial higher operative times and utilization to availability ratios [15]. This has resulted in most robotic training occurring at a consultant career stage rather than during training as it is largely driven by industry and often limited to the private sector. As outlined previously, MIS, including robotics, has opened the door to a range of new educational approaches and opportunities.

## Preceptorship program overview

To address the unique challenges and opportunities within the Australian private sector, this preceptorship program was specifically designed to navigate the landscape of robotic colorectal surgery where access to cutting-edge technology comes up against cost considerations and system integration. In Australia, the adoption of robotic surgery faces unique hurdles such as higher operative costs and slow integration within public health settings. The public availability and uptake where access to a robotic system is available is often hampered by slow business case development limiting who can access it and strict regulation of case selection and application. However, by expanding training, the private environment presents opportunities for innovation and leadership in robotic surgery, driven by a demand for high-quality, minimally invasive procedures. By focusing on these specific challenges and opportunities, this program aims to equip surgeons with not just technical skills, but also the strategic insights necessary for advancing robotic surgery within the Australian healthcare system.

In Sydney, Australia, a colorectal robotic surgery preceptorship program has been developed, targeted at conventionally trained colorectal surgeons. The program is designed primarily for surgeons near to commencing in a consultant role, who have completed sub-specialty training such as the 2-year Australia and New Zealand Training Board in Colon and Rectal Surgery (ANZTBCRS) program leading to Colorectal Surgery Society of Australia and New Zealand (CSSANZ) membership. Candidates need to possess a foundational experience with robotic surgical systems. The program aims to augment their expertise by providing them with advanced exposure to robotic surgical techniques, enhancing their operative competencies in preparation for independent practice. The program is not tailored for trainees who are still awaiting selection for the CSSANZ program. It also does not cater to fellows who anticipate minimal exposure to robotic surgery in their forthcoming consultant roles, as the focus of the preceptorship is on advanced robotic surgical training. It is based in a large private hospital with access to both elective and emergency presentations, leading to a wide range of case exposure.

## Selection

Selection is via local and international advertisement and application. Applicants are ranked based on combined scores for curriculum vitae, cover letter, interview, and references. All applicants must have membership with the CSSANZ or equivalent training to meet scope of practice as a colorectal surgeon (typically 2 years with minimum surgical exposure and logbook evidence, research component, and formal assessment via examination).

## Staged exposure

The program is based on modular learning, divided into three phases, with an additional introductory ‘phase 0’ depending on prior robotic exposure. There is both simulation-based and clinical-based objectives in each phase, with scheduled dry lab and Advanced Robotics courses to be completed approximately mid-way and at completion of the 12 months, respectively. Feedback and progression is based on objective scoring systems, with graded competencies similar to those outlined in the 2014 Clinical Robotic Surgery Association consensus expert roundtable [9]. This rates surgeons as ‘not yet able’, ‘able with assistance’, and ‘able without help’. To progress from one phase to the next, the fellow must not only be competent but proficient in all objectives.

### Phase 0

Phase ‘0’ is an introductory component where prospective fellows are orientated to robotic surgery as required. As robotics becomes more available, more surgeons will have pre-existing awareness and skills that mean this phase is not required but ensures a standardized starting point for phase 1.

Phase 0 objectives are outlined in Table 1.

**Table 1:** Phase 0 components

Objectives	Simulation
General	All modules must achieve score > 90%
Understand advantages and disadvantages of robotics	Camera targeting—level 1
Understand system components	Camera targeting—level 2
Understand patient safety issues	Scaling
Understand patient/operative selection	Complete online intuitive modules (energy use, etc.)
Ability to perform robotic docking	
Safe instrument insertion and exchange	

### Phase 1

This phase aims to introduce hands-on experience as the robotic surgeon and master bedside assisting and provide the fellow with the skills to deal with potential problems. For example, this may involve safely targeting and grasping a bleeding vessel. Fellows must also understand the technology being used and have the ability to troubleshoot from the surgeon console. I begin to learn the use of the robot and performing simple modular components for which they would already be experienced performing in an open and laparoscopic approach. Simultaneously, the program aims to develop the fellow’s ability to become a future competent teacher giving them in-depth knowledge that they can later pass on. This phase may last from 3 to 5 months and incorporates both clinical cases and simulation, additional details are outlined in Table 2.

**Table 2:** Phase 1 components

Objectives	Simulation
Aim to master bedside assisting and have skills to deal with potential problems, i.e., can target to grasp bleeding vessel	All modules must achieve score > 90%
Must understand the technology	Matchboard—level 1
Ability to troubleshoot from console	Matchboard—level 2
Simultaneously develop ability to become a competent future teacher	Matchboard—level 3
General	Dots and needles—level 1
Comfortable with basic instrument manipulation, including camera control and clutching	Dots and needles—level 2
Ensure safe and fluid movement of robotic arms, understand how the robot externally interacts with the patient	Needle targeting
Port placement philosophy and strategy	Ring and rail—level 1
Ability to use visual cues to assess tissue tension	
Proficient use of monopolar and bipolar cautery for dissection and hemostasis	
Clinical	
Fellow as bedside assistant (minimum five cases)	
Mobilization of lateral colonic attachments	

### Phase 2

Phase 2 focuses on intermediate skill development and development of correct technique, progressing problem-solving and early adaptability to variation. Development, progress goals, and feedback are facilitated by the engagement in formal video reviews and practicing specific surgical steps, with a focus on refining techniques for integral surgical procedures such as intracorporeal anastomosis (ICA) and ensuring tension-free tissue exchanges, crucial for patient outcomes. Additional details are outlined in Table 3.

**Table 3:** Phase 2 components

Objectives	Simulation
Proficient in all phase 1 areas	All modules must achieve score > 90%
Become proficient not just in basic operating of the robot but also in the use of robotic adjuncts and settings, e.g., staplers, indocyanine green (ICG)	Pegboard—level 1
Begin to apply this to subspeciality colorectal procedures and build upon general surgery robotic skills	Pegboard—level 2
Focus on performing key steps to achieve outcomes for right- and high left-sided colorectal surgery. This will include ileocolic artery control and division, intracorporeal anastomosis (ICA)	Pick and place
Ongoing focus on tissue exchange without tension	Energy dissection—level 1
General	Energy dissection—level 2
Proficient use of robotic stapling devices	Energy switching—level 1
Proficient in suturing	Energy switching—level 2
Proficient use of the third arm	
Clinical	
Left side colorectal surgery	
Completion of portion of TME rectal dissection	
Completion of majority of left colon mobilization in anterior resection right side colonic surgery	
Completion of majority of ascending colon mobilization	
Completion of entirety of ileocolic intracorporeal anastomosis	
Complete formal video review session	

### Phase 3

This phase is the completion of the fellowship and is based on advanced skill consolidation and ensuring proficiency. Advanced refinement of skills occurs and developing adaptability to unexpected or atypical situations occurs. Late in phase 3, there is a milestone of completing the Advanced Course, signifying the transition from learning to mastery, preparing the fellow for independent robotic colorectal surgery practice. Additional details are outlined in Table 4.

**Table 4:** Phase 3 components

Objectives	Simulation
Phase 3 aims to consolidate and complete training to ensure proficiency as a robotic colorectal surgeon	All modules must achieve score > 90%
General	Stacking challenge
Proficient manipulation of flex joints and 3D dimensional conceptualization and thinking	Ring walk—level 1
Independent case planning	Ring walk—level 2
Efficient use and swapping of master controls	Ring walk—level 3
Proficient exchange of tissue without any undue external or internal collisions	Suture sponge—level 1
Clinical	Suture sponge—level 2
Completion of entirety of TME for rectal dissection and all steps of anterior resection	Suture sponge—level 3
Completion entire right-sided hemicolectomy with ICA; competent in complete mesocolic excision where appropriate	
Complete 20 cases with majority of procedure performed by fellow at robotic console	
Log minimum 35 cases as primary operator by end of academic year	

## Feedback and competency-based assessments

Exposure is predominantly to procedures on the colon and rectum including colonic and rectal resections ranging from right hemicolectomy to ultralow anterior resection. Other common procedures include rectal prolapse surgery, and non-colorectal procedures such as robotic ventral and parastomal hernia repair. Procedure totals are outlined in Table 5 with a goal to complete a minimum 20 cases performing the majority of cases and a total of logging closer to 35 cases in a year. The cognition and integration stages of skill acquisition are already largely completed having completed surgical education and post-fellowship colorectal training, with the focus of the preceptorship on automation (mastering the technical aspects and full benefits of robotic surgery) and the ‘after’ with deliberate practice and coaching from the preceptor—an already proficient robotic surgeon.

**Table 5:** Robotic colorectal procedure case totals and subgroup location

Annual case numbers by academic year	
2021	86
2022	71
2023	120
Total procedures involved with preceptorship program	277
Left-sided rectal procedures	91
Right-sided colonic procedures	57
Other colorectal procedures	59
General surgery hernia procedures	70

Operations are typically broken down into individual stages or modular components, and deliberate practice focuses on each modular component before combining them as a single operation. For example, an ultralow anterior resection would consist of breaking down the well-established process into the following modular components performed robotically:

- Pre-operative special considerations for robotic surgery. Patient suitability and selection, pre-operative anatomical review, and imaging are routinely and formally performed for every patient. Robotic setup, docking, and port placement.
- Mobilization of the colon from rectosigmoid to splenic flexure
- Mobilization of the transverse colon and splenic flexure
- Identification and ligation of the IMV
- Identification and ligation of the IMA
- Rectal dissection in the TME plane with preservation of the autonomic nerves
- Rectal transection and anastomosis

To facilitate discussion and objective review, utilization of case recording occurs and allows for fellow and surgeon mentor to review, discuss, and reflect together. As outlined previously, competency-based objective scoring is used and within each modular component, scores of ‘not yet able’, ‘able with assistance’, and ‘able without help’ are provided.

## Case load and case mix data

A total of 277 cases have been recorded as part of the preceptorship program since inception in February 2021 to January 2024. Annual number of cases the training fellow is involved in is outlined in Table 1 and has ranged from 72 to 120 cases per academic year. Further breakdown by extent of involvement is also included, such as whether the case was observed, whether docking was performed, and/or whether console operating was performed (partial or complete case). Operative category and sub-class are also outlined, with 72% of cases being specialty colorectal surgery cases, including colorectal resections. Other cases related primarily to general surgery procedures such as small bowel pathology or hernias. Mean number of cases spent observing, learning, and performing docking, and developing bedside proficiency prior to console time was 14. Case numbers are outlined in Table 5.

## Outcomes

The unit has a process of continuous interval audit of cases as a department, and current monitoring has shown no significant increase in complication rates. A key component is that surgeons as preceptors are themselves beyond the ‘cognition’ learning stage, and mentors are already beyond the procedure learning curve, and as such, complication rates have remained at a plateau. While numbers are only initial experience and kept for audit purposes, no significant change in adverse outcomes has been noted suggesting the possibility of limiting the impact of a learning curve. An audit of rectal resections undertaken during and prior to the preceptorship program showed the practice leak rate remains below 3%, return to theater remains low (< 1%), length of stay has not significantly changed with a mean of 5.1 days, bloods loss remains an average of 100 ml or less, and while when audited console time slightly increased to a mean of 208 min compared to 187 min prior to the program, this was not statistically significantly (*p* = 0.15). Long-term audit and publication of complication rates is planned following year 5 of the program. So far, the unit has trained three fellows with high annual demand and the role being filled up to 24 months in advance.

## Industry involvement

Significant device and training resources are provided to fellows by Intuitive medical. Initial orientation is performed online, followed by an in-person assessment and setup on the robotic console. Company representatives are able to set up customized learning plans including simulation modules tailored to previous experience. Once complete, a wet lab over 1–2 days can be arranged which allows for advanced practice of skills. Furthermore, fellows aim to be ready for regular dry lab exposure by 6 months. It is important to recall that surgeons undergoing this preceptorship have received the training required to perform the proposed procedures via a laparoscopic and open approach, and mastery of the robotic technique is the goal. As such, the simulations and dry lab allow practice and demonstration of baseline competency and safety in operating the robot rather than robotic proficiency specified technique, and the modular approach to clinical practice allow this to be performed in a controlled and supervised setting.

## Challenges in a private system and benefits for patients

In the private setting, there needs to be buy in from patients. Patients are fully informed and given a choice as to the team members involved. If agreeable, it is explained that as part of the primary surgeons practice, they have a second fully trained colorectal surgeon assisting in the operation, who is developing their skills in the robotic technique. As part of this, they may perform parts of the operation under direct supervision of the primary surgeon. The inclusion of a second surgeon is felt to significantly elevate patient care at no additional cost and is above the standard of care where a surgical assistant is often used that is not a fully qualified surgeon. This dual-surgeon approach mitigates fatigue, a critical factor in maintaining surgical precision and focus. The collaborative synergy between two experienced surgeons also increases the available expertise and perspectives, crucial for complex surgical interventions. This model excels in real-time troubleshooting and problem-solving, addressing intraoperative challenges more effectively. It is explained that perhaps contrary to initial patient apprehensions, the second surgeon is not a novice but is refining and mastering new techniques, as per the Hamdorf and Hall continuum’s ‘after’ stage. This ensures that both surgeons are highly competent, with the preceptorship focusing on advanced skills rather than basic surgical training. The dual console operating system further exemplifies this approach, offering an augmented surgical process with enhanced precision and efficiency with control available to both surgeons. Ultimately, this structure prioritizes patient safety and quality of care, underscoring the commitment to delivering the highest standards of surgical practice.

## Post-preceptorship peer network and robotic mentors

In the rapidly evolving domain of robotic technology, post-training support mechanisms play a crucial role in optimizing the practical application and adaptation of skills. Having completed a preceptorship not only provides technical skills to the surgeon but establishes a support network of colleagues. Lifelong learning and development are a critical component of a career in medicine and surgery. Research consistently underscores the advantages of mentorship and robust peer networks following formal robotic training, and its absence has been shown to be a barrier to implementation of a robotic approach [16].

According to Barrenho et al., who looked at the uptake of laparoscopic surgery in the United Kingdom, younger, female, and internationally trained surgeons were found to be early adopters of laparoscopic surgery [17]. Key attributes of early adopters also included having more professional connections, being situated in teaching hospitals, and having more peers already performing the procedure. The study also underscored the significance of surgeon-level factors in adopting new medical technologies and methods, emphasizing that variation in adoption rates is more pronounced among surgeons than hospitals [17]. The influence of peer networks and connections in the professional realm has also been highlighted—the adoption patterns of surgeons were influenced by those in their network who had already embraced the technique, especially pioneering adopters [18].

A mentor or peer network provides invaluable personalized guidance, feedback, and support, helping the less-experienced surgeon navigate through complexities and unanticipated challenges in the real-world application of robotic technologies. This mentor–mentee relationship bridges the gap between theoretical learning and practical execution, facilitating quicker problem-solving and refinement of techniques. Moreover, peer networks create a collaborative learning environment, fostering the exchange of ideas, experiences, and innovative solutions beyond the initial preceptorship. Such interactions catalyze continuous learning and adaptation increasing utilization of a robotic approach. Collectively, mentorship and peer networks not only enhance the efficacy of the training but also contribute to the ongoing development, resilience, and excellence of professionals beyond the formal period of preceptorship.

## Conclusion

This preceptor program in the Australian private sector provides an invaluable and unique opportunity for colorectal surgeons to gain proficiency as robotic colorectal surgeons. This program is positioned to provide improved patient care while also facilitating the transfer and mastery of robotic colorectal surgery through established surgical education techniques.

## Data Availability

No datasets were generated or analyzed during the current study.
